# Intelligent judgements over health risks in a spatial agent-based model

**DOI:** 10.1186/s12942-018-0128-x

**Published:** 2018-03-20

**Authors:** Shaheen A. Abdulkareem, Ellen-Wien Augustijn, Yaseen T. Mustafa, Tatiana Filatova

**Affiliations:** 1 Department of Governance and Technology for Sustainability (CSTM), Faculty of Behavioral, Management, and Social Sciences (BMS), University of Twente, Enschede, The Netherlands; 2 Department of Computer Science, College of Science, University of Duhok (UoD), Duhok, Kurdistan Region Iraq; 3 Department of Geo-Information Process (GIP), Faculty of Geo-Information Science and Earth Observation (ITC), University of Twente, Enschede, The Netherlands; 4 Faculty of Science, University of Zakho (UoZ), Duhok, Kurdistan Region Iraq; 5 School of Systems, Management and Leadership, Faculty of Engineering and Information Technology, University of Technology Sydney, Ultimo, Australia

**Keywords:** Protection motivation theory, Disease diffusion, Emergent behavior, Learning, Cholera, Bayesian networks

## Abstract

**Background:**

Millions of people worldwide are exposed to deadly infectious diseases on a regular basis. Breaking news of the Zika outbreak for instance, made it to the main media titles internationally. Perceiving disease risks motivate people to adapt their behavior toward a safer and more protective lifestyle. Computational science is instrumental in exploring patterns of disease spread emerging from many individual decisions and interactions among agents and their environment by means of agent-based models. Yet, current disease models rarely consider simulating dynamics in risk perception and its impact on the adaptive protective behavior. Social sciences offer insights into individual risk perception and corresponding protective actions, while machine learning provides algorithms and methods to capture these learning processes. This article presents an innovative approach to extend agent-based disease models by capturing behavioral aspects of decision-making in a risky context using machine learning techniques. We illustrate it with a case of cholera in Kumasi, Ghana, accounting for spatial and social risk factors that affect intelligent behavior and corresponding disease incidents. The results of computational experiments comparing intelligent with zero-intelligent representations of agents in a spatial disease agent-based model are discussed.

**Methods:**

We present a spatial disease agent-based model (ABM) with agents’ behavior grounded in Protection Motivation Theory. Spatial and temporal patterns of disease diffusion among zero-intelligent agents are compared to those produced by a population of intelligent agents. Two Bayesian Networks (BNs) designed and coded using R and are further integrated with the NetLogo-based Cholera ABM. The first is a one-tier BN1 (only risk perception), the second is a two-tier BN2 (risk and coping behavior).

**Results:**

We run three experiments (zero-intelligent agents, BN1 intelligence and BN2 intelligence) and report the results per experiment in terms of several macro metrics of interest: an epidemic curve, a risk perception curve, and a distribution of different types of coping strategies over time.

**Conclusions:**

Our results emphasize the importance of integrating behavioral aspects of decision making under risk into spatial disease ABMs using machine learning algorithms. This is especially relevant when studying cumulative impacts of behavioral changes and possible intervention strategies.

## Background

Globally, millions of individuals are regularly exposed to deadly infectious diseases. For example, news of the Zika virus outbreak was one of the main news stories of the past 2 years. Perceiving disease risk motivates people to adapt their behavior toward a safer and more protective lifestyle. Indeed, risk perception (RP) is an integral part of the decision-making process under uncertainty and can be understood as an individual’s evaluation of risk in a particular situation. This evaluation includes individual assessments of how severe and controllable a particular situation is. The reliability and effectiveness of any risk evaluation by an individual is based on the risk information available [1]. Accordingly, the availability of risk information impacts the perception of a decision problem, the evaluation of available options, and of any risk-coping decisions [2]. A number of factors related to the design of a risk message influence risk perception: the message, being the source of information (other people, and/or the environment), and the adaptive behavior in response to that message. These factors need to be considered in order to design effective risk communication strategies and to positively influence health-related decisions [3].

Numerous examples of human behavior influencing the spread of infectious diseases are available [4]. Namely, Manfredi and D’Onofrio (2013) refer to human behavior as to the neglected layer of complexity in current epidemiological models [5]. In the latter, the response to risk factors is fixed, and no effect of previous exposure—or learning—is incorporated in most models. This implies that a disease model may underestimate the effectiveness of preventive measures. This can lead to a higher scope of contagion compared to a real situation, consequently leading to an overestimation of the prevalence of disease cases. Instead, employing learning techniques to capture dynamics in RP and corresponding protective behavior can mimic the complex process of how human beings act upon encountering risk.

Behavioral science has developed various theories to explain, measure, and assess RP. Protection motivation theory (PMT) is one of the dominant approaches in this domain, and has already been applied to the study of health-protective behavior [6]. Originally proposed by Rogers [7], PMT has been actively applied in health research to study cognitive processes and predict health-related behavior. Behavioral aspects of decision-making under risk are active with ABMs [8–10] outside disease of research, and often without facilitating learning. In fact, ABMs are instrumental in exploring and implementing RP, such as the risk of disease diffusion. Disease ABMs have become significantly sophisticated by integrating rich GIS landscapes with detailed human activities (e.g. mobility and social networks) as well as multi-stage epidemiology models such as the SEIR (Susceptible–Exposed–Infected–Recovered) model. Moreover, ABMs are able to incorporate the social behavior of individual agents as well as the dynamics of the spatial environment, which also plays an important role in the disease diffusion process. Various infectious diseases have been modeled using ABMs [14–16]. Wise [14] provides an extensive review of disease and disaster ABMs. Although ABMs are technically suitable for incorporating agents with higher levels of intelligence, this is rarely implemented in disease models. For example, RP typically enters decision-making models either as a variable affecting a decision-making process or as a step within a rule-based procedure [15–18].

In rule–based implementations, behavior is fixed, meaning that decision-making functions and algorithms remain unchanged. While agents react to changes in their spatial and social environment, they neither adapt their rules in response nor intelligently learn from previous experiences. This is unrealistic, as human beings adjust their behavior strongly when they perceive a serious risk, which can potentially lead to disease models overestimating risk. Intelligence helps agents assess risks and potentially adapt their behavior—i.e. learn to reduce or avoid health risks—based on changes in RP.

To test the impact of adaptive RP in human decision-making, we implement PMT in a spatial disease ABM. Namely, we extend the base disease model developed by Augustijn et al. [19] to the behavioral aspects of decision-making in a risky situation using machine learning (ML) techniques. The spatial agent-based disease model—Cholera ABM—is applied to study the spread of cholera in Kumasi, Ghana. In this article, we use Bayesian Networks (BNs) as the learning method to design intelligent agents behaving according to PMT and making decisions on how to cope with cholera in a rich spatial environment. We systematically test the impact of intelligent behavior on disease spread through a series of simulation experiments: using Cholera ABM with zero-intelligent agents, agents enhanced with ML for updating their RP, and agents enhanced with ML for RP and coping appraisal behavior dynamics. BNs replace ad hoc rule-based schemes for uncertainty reasoning due to their capability for bi-directional inference combined with a strict probabilistic foundation [20]. They are capable of sensing and reacting to a stochastic environment. In addition, BNs have the ability to constantly adjust to simulate the dynamics of agents’ beliefs. Therefore, BNs have been implemented in ABMs as the agents’ cognitive model for different purposes, including negotiation [21], prediction [22], and adaptation [23].

## Methods

We start by briefly describing the base ABM and then focus closely on the describing the learning algorithms and their stepwise implementation to support agents’ intelligence.

### The base cholera model and zero—intelligence agents (ZI)

**Fig. 1:**
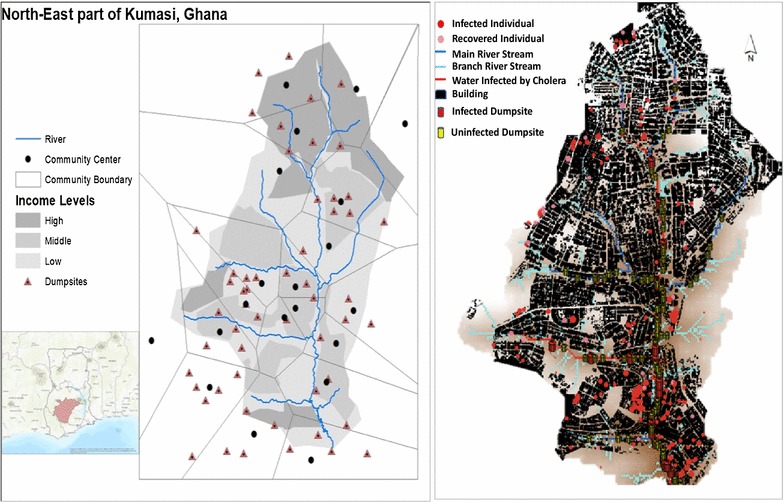
Left hand: study area with community boundaries: we used Thiessen polygons to define the boundaries of communities that were unknown or ill defined. Right hand: Spatial spread of cholera in a typical simulation

The Cholera ABM is used as a testbed for this research. The model was developed to test if runoff water from open dumpsites could have been the diffusion mechanism behind the 2005 cholera outbreak in Kumasi Ghana. This ABM simulates both a hyper-infectious and a low-infectious diffusion route of cholera. It is a spatial ABM with a rich representation of GIS data, including elevation, the location of residential areas, river hydrology, and the location of dumpsites in the study area (Fig. 1).

**Fig. 2:**
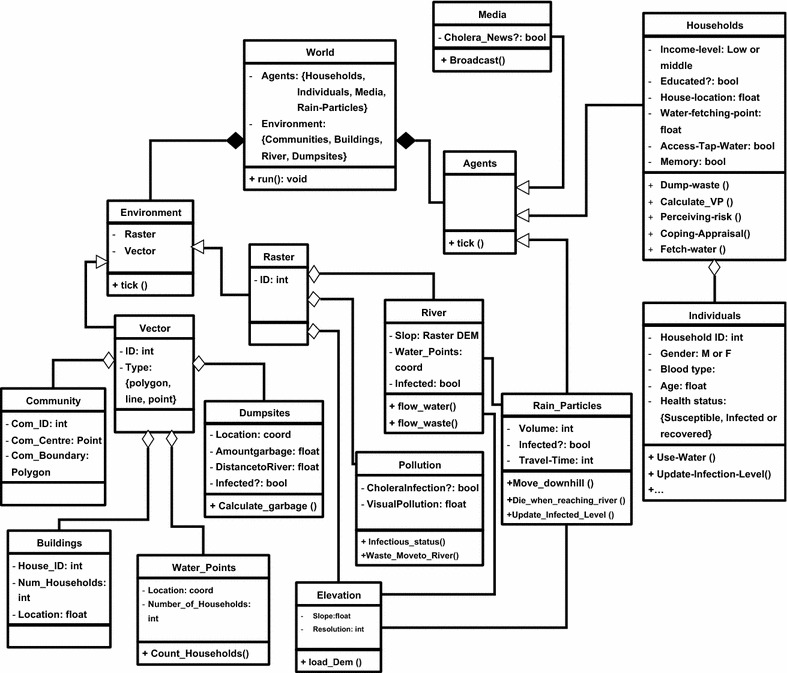
The UML diagram of Cholera ABM

The Cholera ABM contains three types of agents: households, individuals, and rain particles (Fig. 2). The model contains three sub-models: a *hydrological mode*l, an *activity model*, and a *disease model*. The hydrological model moves rain particles over the area. Following heavy rainfall, runoff water can become infected with cholera bacteria when passing through dumpsites, thereby transporting cholera bacteria into the river. Via the activity model, household agents will determine the type of water they should consume (tap water, bottled water, or river water).

Household agents use river water when tap water is unavailable. When a household agent uses river water, the model will choose the river location closest to agent’s home and determine if the water at this location is infected. Individuals can become infected by using water polluted with cholera and will subsequently shed hyper-infectious materials that will be dumped by the household to the nearest open refuse dumpsite. This increases the infection level of this dumpsite and the probability of rain particles becoming infected. Finally, the disease model will determine the progression of the disease in the individual and the moment of recovery. However, this Cholera ABM does not include cholera RP and behavioral change (the selection of another water source) of agents—i.e. the household agents have no intelligence. They follow the same behavior and activities during the entire simulation period. The time step of the model is 1 h, with a time horizon of 90 days.

### Intelligent agents: how do intelligent households make decisions?

#### Protection motivation theory (PMT)

**Fig. 3:**
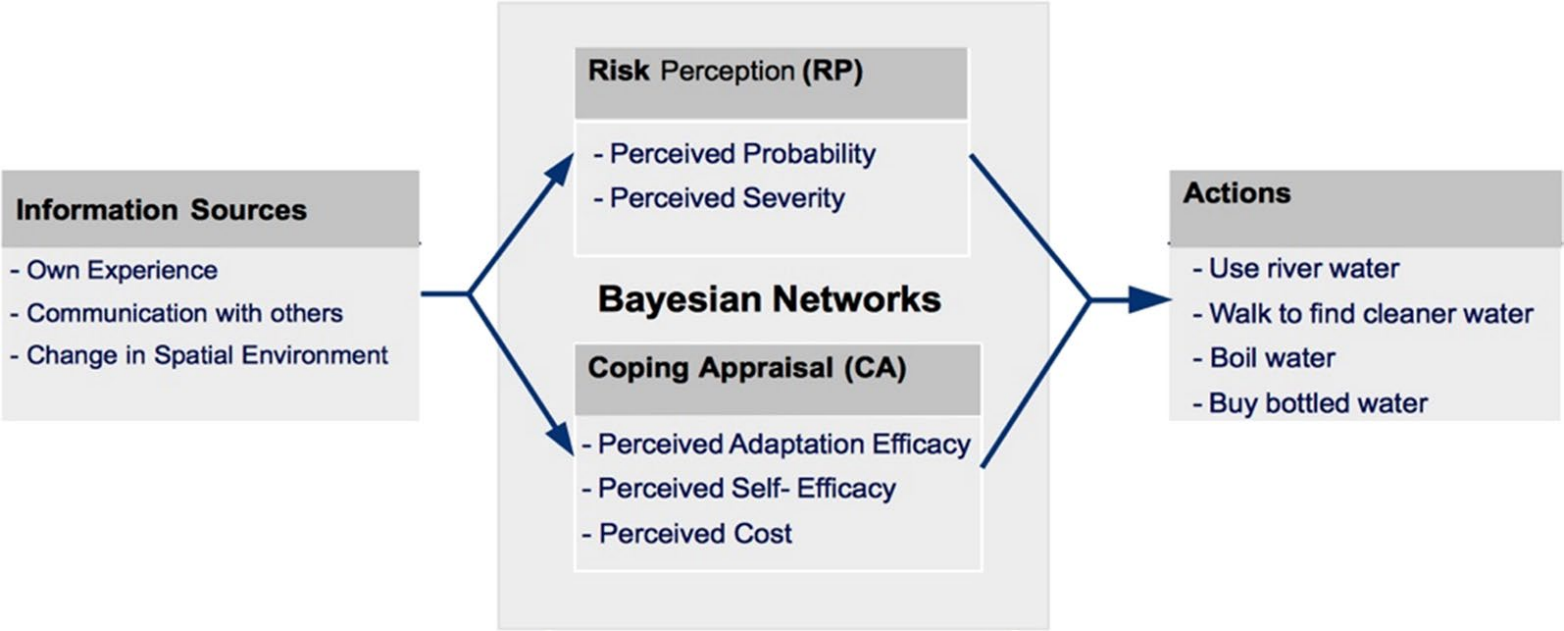
Cognitive process of protection motivation theory (PMT)

PMT is used as the theoretical framework of this paper. PMT considers that, when facing a risky situation, a person goes through two steps: “threat appraisal” and “coping appraisal” (Fig. 3). Threat appraisal in PMT is the stage at which perceptions of risk are formed. Here, a household agent assesses the probability and consequences of a risky event occurring—i.e. perceived probability and perceived severity, which in fact constitutes the agents RP. Therefore, in the proceeding sections of this paper we refer to threat appraisal as the stage at which RP is developed. The perception of severity enables households to judge how seriously the consequences could be, should they face a threat. Perceived probability measures how susceptible a person is to a given threat. The purpose of this stage is to detect whether a risk is at an acceptable level or not.

When RP is sufficiently high, household agents consider a number of protective behaviors by passing through the coping appraisal stage. The coping stage consists of two main parts: adaptation-efficacy and self-efficacy. Adaptation-efficacy measures the effectiveness of protective behavior against a harmful situation—i.e. the beliefs of a person that the recommended behavior will protect them. Instead, self-efficacy measures the ability of a person to perform the recommended behavior. In addition, the person must evaluate the cost of coping with the threat. Hence, at this stage, households consider the psychological, physical, and economic consequences of adapting to a particular threat.

#### Cholera ABM—intelligent agents

In the intelligent version, the Cholera ABM is modified to simulate the RP (threat appraisal) and coping appraisal (CA) processes of household agents—i.e. including the learning technique to create intelligent agents. For this purpose, one extra agent (media) is added to the model (Fig. 2).

The state variable of the *Household agent* is the type of water they consume, and the infection level of this water. The household agent is responsible for the collection of water, and all household members will use this water for their daily consumption. Learning takes place at the level of the household, as it is directly related to the water source that the household selects. To facilitate this learning, we added memory and education level to the attributes of the household agent.

The state variable of the *Individual agent* is their health status. Individual agents can be susceptible to, infected with, or recovered from cholera.

Some studies have indicated that medical alerts do not have the impact of encouraging people to physically search for medical advice during epidemics [24]. However, information received from different media channels can prevent an epidemic from spreading [25]. Therefore, *Media* is a new agent that has been introduced to broadcast information about the epidemic in this model. The state variable of the media agent is its activation level, which determines if the media agent has started to broadcast about the epidemic.

The state variable of the *Rain particles* agent is the infection level. While flowing over the terrain, rain particles can acquire the infection (from infected dumpsites) and carry it to the nearest river or tributary.

The processes included in the original model were flow of rain particles, household fetching water, and households dumping their waste. These processes remain unchanged in the version of the model used in the present research. However, in this version of the cholera model, we added the following processes:

1. Activation of the media agent;
2. Clearance of the dumpsites;
3. Calculations of the visual pollution (VP) level;
4. Risk perception;
5. Coping appraisal (CA).

## Activation of the media agent

The media agent is deactivated in the beginning of the simulation. It is activated when the number of days exceeds a threshold value (22 days). After activation, the media agent will broadcast news about the cholera epidemic once a day, which all household agents in the simulation will receive. Once the broadcasting has begun, it will continue throughout the remaining part of the simulation. Media information is used in the risk assessment.

## Clearance of the dumpsites

In the original model, dumpsites could be infected with cholera, and when the decay function was activated, this infection would gradually disappear over time. We also introduce the fact that garbage will be removed from dumpsites. This has two separate effects: it will influence the infection and will also have an impact on the visual pollution level.

Clearance of dumpsites will occur randomly. In Kumasi, 85% of household waste is collected by the municipality from the dumpsites twice per week [26]. Therefore, in this model, a random 85% of simulated dumpsites are discharged twice per week.

## Calculation of the visual pollution level (VP)

Household agents fetch water from the nearest water collection point on the river, either because they do not have access to tap water, or because their tap water has stopped working due to heavy rain. Open refuse dumpsites are located at varying distances along the river. It is common in Kumasi to observe waste dumps located on riverbanks or in a river’s path [27]. In the simulation, risk will be assessed based on a combination of factors, including the visual pollution (VP) level of the water collection points. The visual pollution level is calculated based on the combined link order and the number of open refuse dumpsites located within a specific distance from the river. VP is calculated based on the following equation:

1 $${\text{f}}\left( {\text{VP}} \right) = \mathop \sum \limits_{{{\text{i}} = 1}}^{\text{N}} \frac{{{\text{x}}_{i} {\text{g}}_{i} }}{{{\text{d}}_{i} }}$$

where N is the number of dumpsites around the water collection points; x_i_ is the number of households who use the dumpsite; g_i_ is the amount of garbage produced by each household; d_i_ is the distance from the dumpsites to the water collection point; and i represents all dumpsites in N (either cleared or not). Although the number of dumpsites is fixed throughout the simulation, the amount of garbage remains static, and the number of households will also remain static over a simulation run, while the visual pollution level is dynamic. This dynamic nature is due to the random selection of dumpsites that will be cleared over a simulation run.

## Learning—implementation of agent’s cognitive model

**Fig. 4:**
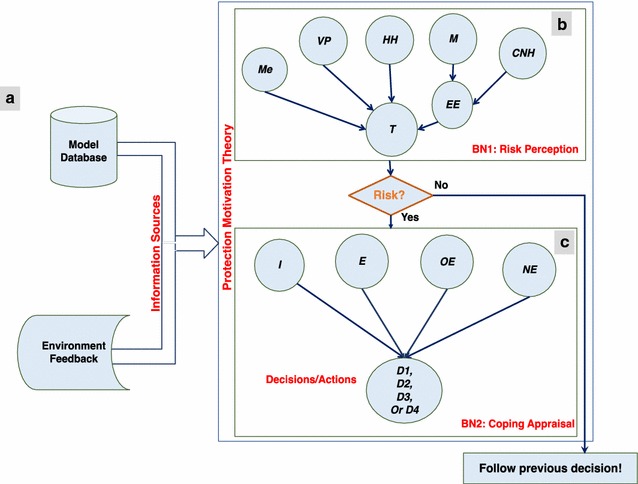
Implementation of PMT: **a** information sources; **b** BN1 (RP); **c** BN2 (CA)

The PMT drives the agents’ cognitive model. The information sources and the two stages of PMT are illustrated in Fig. 4. In this model, we used two BNs – BN1 to model the RP, and BN2 to model the CA.

### Implementation of risk perception (RP)

At each time step, the household agent will perceive the risk of cholera infection using the BNs. The following factors are included in the RP: the number of infected individuals in the household, visual pollution level at the water collection point, communication with other agents, media attention, and the memory of the household agent. Together, these factors and the agents’ social interactions help agents to assess risk and thus select what decision they could make among several options.

#### Communication with other agents (social networks)

Household agents are assumed to have a total awareness of the cholera cases occurring within their neighbors’ subset. A neighbor is defined as a household agent, sharing the same water collection point and living in the same community. Interaction with neighbors enables agents to perceive the infection level of the water collection point they use. In addition, household contacts help agents to gain information on adaptive decisions their neighbors took and how effective these decisions were.

No data is available on how many daily contacts Kumasi residents have. However, in a recent study by Melegaro et al. [28], they conducted a survey of daily contacts in Manicaland, Zimbabwe and reported 10.8 contacts per person/day, including contact with household members. If we consider this rate for our study and exclude the number of household members (average of 3.9), then approximately seven contacts with neighbors per day should be applied. These seven neighbors are chosen randomly every day from the agent’s community.

#### Memory

Agents use their memory to record the RP they experienced during the previous day (the last day they fetched water) and how preventable their last decision was. The feedback of the last decision made is measured by “positive experience” if no illness was observed in the household, otherwise it is a “negative experience”.

#### BN1: risk perception

BN1 was designed to represent the RP of PMT in such a way that it answers the question “is there risk?” In the case of a risk being present, agents will proceed to the CA.

Agents with a low or medium income level that do not have access to safe water will fetch water from the river. Therefore, they must evaluate the risk of becoming ill with cholera using BN1. In our case, BN1 is formed by the cause-and-effect concept. To design BN1, we derive five nodes from the information sources to evaluate RP (Fig. 4b). These nodes include: *memory* (*Me)*, *visual pollution (VP)*, *household health status (HH)*, *media (M)* and *communication with neighbor households (CNH)*. Media and communication with neighbor households are combined into “Epidemic Evidence” (*EE)*. *EE* is a binary measure that indicates to the agents if there are cholera cases outside their own households. The evaluation of infected cases differs by agent due to variations in household income and size, in the health status of different households, in their locations within the city that define VP and their selection of neighbors with whom they communicate, and in the experiences stored in their individual memories.

The reasoning and uncertainty of RP is governed by rules that can be formalized using formula (2). For example, we include the states {*yes*, *no*} for *memory (Me)*, {*yes*, *no*} for *threat (T)*, then the formula of connecting these two variables accordingly was designed as:

2 $${\text{P}}\left( {T_{{\left\{ {yes, no} \right\}}} |Me_{{\left\{ {yes,no} \right\}}} } \right) = \frac{{{\text{P}}\left( { Me_{{\left\{ {yes, no} \right\}}} |T_{{\left\{ {yes, no} \right\}}} } \right){\text{P}}\left( {T_{{\left\{ {yes, no} \right\}}} } \right)}}{{{\text{P}}Me_{{\left\{ {yes, no} \right\}}} }}$$

in such a way that each state of *Threat* is examined with each state of *memory*.

This was also applicable for computing the probability (P) of *threat* based on *visual pollution (VP)* and *household health status (HH),* as both variables have the states {*high*, *low*} and {*yes*, *no*}, respectively.

We evaluated the epidemic evidence (*EE)* that agents record via their communication with neighbor households (*CNH*) and the media (*M*) agent.

According to Bayesian rules, the prior probabilities of the nodes should be specified in order to gain the posterior probabilities. These prior probabilities represent the integral part of human reasoning regarding certainty. The prior probabilities will be updated/changed for each agent on the basis of information being passed by each agent to BNs. In BNs, this is called evidence.

The final formula for the *threat* node (*T*) that derives the conditional probability table (CPT) will depend on *memory (Me), visual pollution (VP), the health status of household (HH),* and the *severity evidence of epidemic (EE)*:

3 $$P\left( {T|Me, VP, HH,EE} \right) = \frac{{P\left( {Me, VP, HH, EE|T} \right)P\left( T \right)}}{{P\left( {Me, VP, HH,EE} \right)}}$$

Thus, intelligent agents in the Cholera ABM learn to predict health risks with the help of BN1 (Eq. 2). In BN1, the memory node feeds the network with previous information on agents’ own RP. Agents learn to revise their beliefs by absorbing other factors from their environment that are updated during the simulation, e.g. currently observed visual pollution, number of illnesses among neighbors, etc. (Eqs. 2–3). Agents conclude the causal relationship between nodes in the BN1 by inference. The output of BN1 would be the probability of high or low risk perception. We consider the agent to be at risk if the probability of RP is greater than or equal to 0.5.

### Coping appraisal (CA)

BN2 was designed to represent the coping appraisal of PMT in such a way that it answers the question “what to do?” In the case of perceiving risk, an agent may either: use the polluted water anyway, walk (find another location to fetch water), boil the fetched water (to increase safety), or purchase bottled water. To select one of these four decisions, a number of variables (nodes) affecting this process were identified and used. These variables include: the income level of the agents (medium or low); their education level (educated or uneducated); and the feedback of their previous and their neighbors’ previous action (positive or negative). Agents cannot learn from their own experience unless they have a feedback on their previous actions [29]. Together, all of these dynamics guide the decision-making process.

#### BN2: coping appraisal

BN2 represents the structure of the CA (Fig. 4c). The probability of which decision might be chosen by the agent is computed via BN2. The perceived adaptation efficacy will differ per decision. Walking to another location to collect water has a lower efficacy compared to boiling the water, and this has a lower efficacy compared to buying bottled water. Also, perceived self-efficacy (i.e. perceived effectiveness enabling an agent to perform the preventive measure) is varied for each decision. In addition, the perceived costs of the options differ, as river water is free of cost, boiling water has a price tag, and so does the purchase of bottled water. Here, the agents’ income level determines which decision is more likely to be taken.

The formula of BN2 for computing the CPT of a decision can be expressed as:

4 $$P\left( {D|I, E, OE, NE} \right) = \frac{{P\left( {I, E, OE, NE|D} \right)P\left( D \right)}}{{P\left( {I, E, OE, NE} \right)}}$$

where *D* stands for decision, which can take the form (state) of ‘use water from the same fetching point’ (*D1*), ‘walk to another fetching point’ (*D2*), ‘boil water’ (*D3*), and ‘buy water’ (*D4*); *I* denotes an income level, what can be middle or low; *E* is the education level (educated or not); *OE* is an agent’s own experience with cholera, which can be either positive (no household member is ill) or negative (at least one household member is ill); and *NE* is the neighbor’s experience with cholera [anyone ill (negative) or not (positive)].

### Model parameterization

The probability values of both networks variables are derived from the existing literature and census data for Kumasi. The census data of Kumasi, Ghana includes income distribution. The distribution of the three levels is 19% (low), 52% (medium), and 29% (high). However, we exclude high level incomes since they will not use river water. Therefore, by scaling both medium and low-income levels, we get 73 and 27%, respectively (which represents 71% of the number of simulated households). Additionally, 14% of low and middle-income level households do not have access to tap water. Table 1 presents the additional parameters of this cholera model. Naturally, for real policy application, the quality of data regarding initial weights in BN1 (Table 2) and the frequency and the extent of information delivery, either via media or through the word-of-mouth across social networks, is essential. We run a sensitivity analysis of final outcomes on the initial weights of both BNs (“Appendix 1”). The results indicate that the model is rather robust, with minimal impact on the final outcomes.

**Table 1:** Cholera ABM new parameters

New parameters	Value	Description
Literacy rate	74.1%	[30]
Media	Activation day 22	During the 2005 outbreak, newspapers and TV channels published news about the cholera in the region after about 3 weeks of epidemic started (visit: Ghana News Archive)
Waste collection	85% of dumpsites	85% of waste is collected by Kumasi municipality [26]. The rest remain uncollected for a week or more
Amount of garbage	2.925 kg/household/day	Derived from literature [31]
Number of contacts with neighbors	7 neighbors	Derived from literature [28]

**Table 2:** Model settings varied across the three experiments

Model settings	Exp1	Exp2	Exp3
Threat appraisal Initial weights^a^ *Me*, *VP*, *HH*, *M*, *CNH* Weights during a simulation Outcome	None n.a. n.a. n.a.	BN1 (0.1; 0.2; 0.01; 0.01; 0.2) Change as agents learn RP, (0;1)	BN1 (0.1; 0.2; 0.01; 0.01; 0.2) Change as agents learn RP, (0;1)
Coping appraisal Initial weights *I*, * E*, *OE*, *NE* Weights during a simulation Outcome	None n.a. n.a. D1	Deterministic Rule based, Table 3 Static D1-D4: fixed population share	BN2 (0.52; 0.74; 0.9; 0.6) Change as agents learn D1–D4: adaptive, based on previous experience

^a^To elicit the factors that may play a role in the context of a water-spread disease in a developing country as well as their relative importance we ran a survey among students. We approached the participants of the Massive Open Online Course (MOOC) on GeoHealth run at ITC (authors host institute) in Sep, 2016. Majority of the participants of this course are from developing countries. Ideally, one would survey real citizens in the case-study area. This was not possible due to the lack of funds and access to the potential respondents

## Simulation results

### Experiment setup

**Fig. 5:**
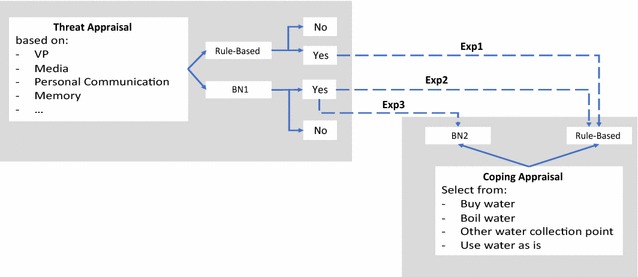
Implementation of PMT in Cholera ABM where Exp1, Exp2 and Exp3 refers to experiments 1, 2, and 3 respectively

To answer the research questions, we have designed three experiments. We systematically vary the cognitive abilities of agents by gradually adding intelligence by means of the two BNs (Fig. 5). In particular, the first experiment (Exp1) presents a benchmark case to study disease diffusion patterns in a spatial landscape with a population of zero-intelligence agents. Agents are heterogeneous in income, education, and household size but have no cognitive abilities to either perceive risk or act upon it. In the second experiment (Exp2), agents are enhanced with the BN that represents the first stage of decision-making in a risky context: the risk appraisal (BN1). As agents learn and interact with each other, the probabilities of specific factors influencing risk appraisal change. The second stage of decision-making in Exp2 is modeled in a simplistic manner by adopting a rule-based algorithm, which deterministically guides an agent to a specific action if its RP is high. Finally, the third experiment (Exp3) adopts intelligent decision making at both stages of decision making under risk: the risk appraisal (BN1) and the coping appraisal (BN2) both supported by BNs learning algorithms. Thus, if agents begin to perceive risk as an outcome of BN1, they employ BN2 to decide how to act upon it. As agents learn from their own experience and others’ through interaction, the probabilities of specific actions to be chosen through BN2 evolve. All other settings among the three experiments remain static (Table 2). Each of the experiments is run 100 times to assure the robustness of the results.

We report the results per experiment in terms of several macro metrics of interest: epidemic curve, RP curve, and decision type curve. An *epidemic curve* is a graphical description of the number of illness cases by date during an outbreak. It illustrates the temporal trend and periods of disease incubation. A *RP curve* is a graphical description of a number of agents that perceive disease threat, i.e. have their RP equal to 1 in a specific time step. A *decision types curve* counts the number of agents following a particular decision when deciding on how to cope with cholera risk. In addition, we show several maps illustrating the spatial patterns of RP (Decisions: D1-D4).

### Disease diffusion in a population of zero-intelligent agents

**Fig. 6:**
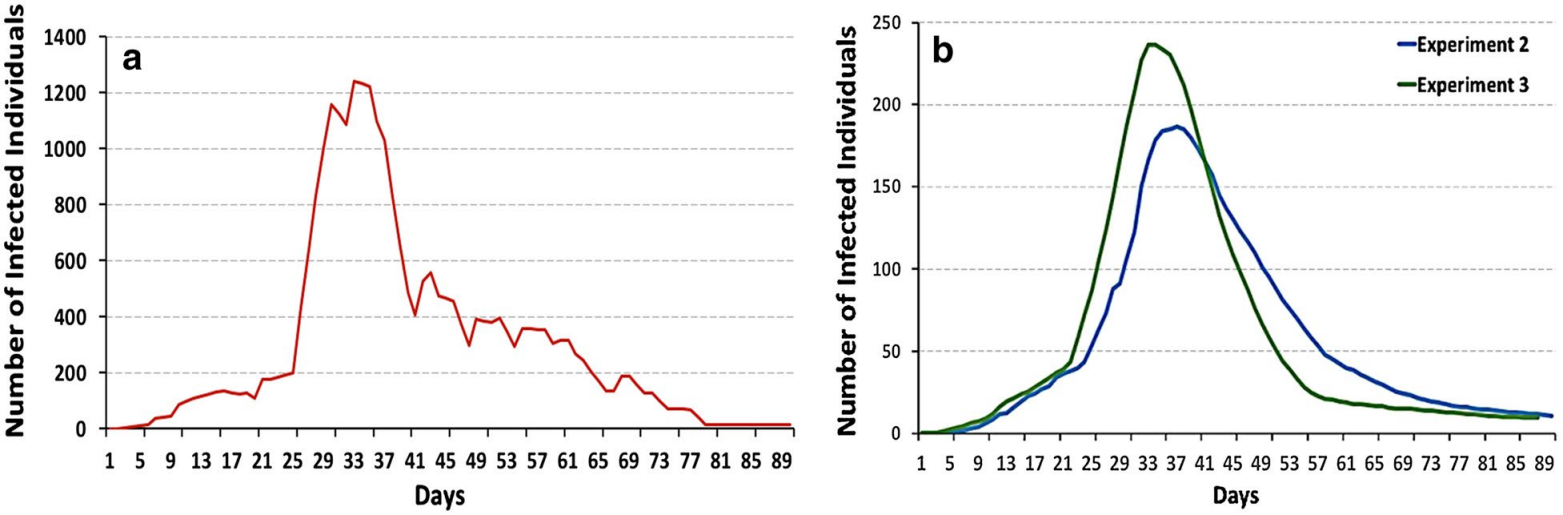
Epidemic curve of Exp1,2 and 3. Average number of infected per time step across 100 runs. **a** Exp1 (zero-intelligent population), **b** Exp2 (blue) and Exp3 (green)

The temporal patterns of a cholera epidemic given a population of zero-intelligent (ZI) agents neither perceiving risk nor pursuing any protective measures is presented in Fig. 6a. It is evident that, even if a household member becomes ill, media broadcasts cholera being present, and some visual pollution is observed at a water fetching point, a ZI agent will still continue to collect water for its daily needs at the same water fetching point and will use it without precautionary measures. The number of infected agents reaches a maximum between day 28 and day 40 before gradually decreasing towards the end of the epidemic. In total, 81% of the simulation population (27,000 out of 34,000 individuals) is infected with cholera in Exp1. While the ZI Cholera ABM succeeds in reproducing the qualitative pattern of this Cholera epidemic, it largely overestimates the number of infected individuals. A simulation with non-adaptive ZI agents misrepresents reality, since even middle income and educated people continue to consume potentially contaminated water: 28.6, 64.7, and 6.5% in the low, middle, and high-income categories, respectively.

When agents have no cognitive abilities, and are not reactive, then the probability of becoming infected during a rainy period depends on the concentration of infected agents, which may dump infected waste on a dumpsite, leading to flow of cholera-infected rainwater into the river.

### Intelligent risk perception

From a psychological perspective, to be able to act upon risk, people—i.e. agents in the Cholera ABM—must first be aware of a risk. Experiment 2 presents the case when intelligence is added in the threat appraisal (BN1) stage. When being aware of risk while fetching water, agents in Exp2 may change their behavior using a deterministic rule-based algorithm (Table 3). Thus, actions that agents select in this CA stage are based on current information, ignoring any previous experiences. Enhancing agents with cognitive abilities for threat appraisal (BN1) reduces the total number of infected agents by 90%. In Exp2, the total number of cholera-infected agents decreases (see the blue epidemic curve of Exp2 in Fig. 6b). In other words, information about a disease spreads through different channels—media, own observations, the experience of others, while a simple set of precautionary actions give rise to a steadier epidemic curve. Following the epidemic peak, agents are risk-aware and take a variety of precautionary actions based on their income class and education, ill individuals in their own and/or their neighbors’ households; thus, fewer infections occur at the later stages of epidemics. Therefore, the BN1 epidemic curve (in Fig. 6b) has a lower peak and a steeper, vanishing tail compared to the ZI epidemic curve (Fig. 6a). The first heavy rainfall boosts the spread of cholera and can be detected in the shape of this curve at approximately day 23 in Exp2. Then, the effect of new disease exposure on the number of infected is counterbalanced by the activated risk awareness within the BN1 population. New exposure occurs when agents either lack infection experience in their social network or choose to ignore risks at the coping stage. The Cholera ABM enhanced with BN1 for the threat appraisal may be used to explore the spatial and temporal patterns of disease spread depending on varying risk communication strategies. To demonstrate this notion, we run a sensitivity analysis on the main communication channels.

**Table 3:** Rule-based algorithm (CA) for Experiment 2 where agents select a static decision to take based on their characteristics

Household characteristics				Decision
Income	Educated	Infection in household	Infection in neighbor households	
Low	No	No	No	D1 (same)
Low	No	No	Yes	D1
Low	No	Yes	No	D2 (walk)
Low	No	Yes	Yes	D2
Low	Yes	No	No	D1
Low	Yes	No	Yes	D2
Low	Yes	Yes	No	D2
Low	Yes	Yes	Yes	D2
Middle	No	No	No	D1
Middle	No	No	Yes	D2
Middle	No	Yes	No	D4 (buy)
Middle	No	Yes	Yes	D4
Middle	Yes	No	No	D1
Middle	Yes	No	Yes	D3 (boil)
Middle	Yes	Yes	No	D3
Middle	Yes	Yes	Yes	D4

#### Sensitivity analysis on the number of social interactions

**Fig. 7:**
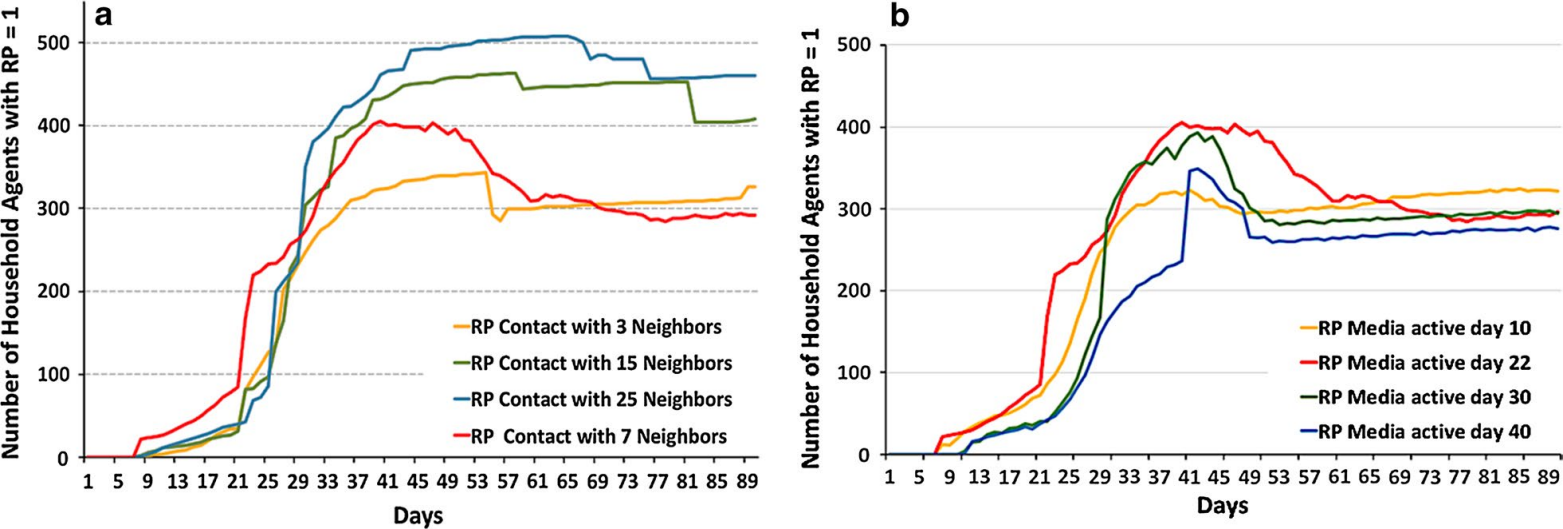
Sensitivity analysis of the risk perception dynamics in a population of BN1 agents (Exp2). Average risk perception curve across 25 runs. **a** Depending on the intensity of social interactions, **b** depending on the timing of the media activation

A diffusion of information about disease risk and the effectiveness of risk-coping measures occur through social interactions. Their intensity impacts the spread of awareness about cholera risk in the study area as well as the number of infected individuals. Following Melegaro et al. [28], the base scenario of Exp2 (and Exp3) assumes that when fetching water, agents exchange information daily with seven agents from their social network. These social links are set up randomly among households in the same community using the same water collection point. In addition, we run sensitivity analysis considering 3, 15, and 25 unique social interactions with individuals outside their own household per day. Figure 7a and Table 4 illustrate the sensitivity of the number of individuals perceiving cholera risk and the resulting number of infections under various assumptions regarding social interaction.

**Table 4:** Sensitivity of the extent of an epidemic on the intensity of social interactions and information exchange among intelligent agents (Exp2)

No. of contacts	RP peak day	Epidemic peak day	Percentage of total population infected from the base (%)
Three	83	35	103
Seven (base)	40	36	100
Fifteen	71	35	75
Twenty-Five	66	36	74

All curves in Fig. 7a demonstrate a steep increase in risk perception around day 23 of the simulations. This point indicates the first heavy rainfall, when the population of agents depending on river water increases, and the disease diffusion via the dumpsites begins. During this first period, all scenarios exhibit the same pattern. However, after day 40, a clear difference is observed between the four scenarios. As expected, the higher the number of daily contacts (with which intelligent BN1-agents exchange information), the higher the number of households who perceived risk. Higher levels of cholera risk awareness trigger agents to make alternative decisions regarding water use (D2-D4 instead of D1), following the deterministic rule-based algorithm, and thus leads to a reduction in the number of infected individuals (Table 4).

With fewer social interactions, BN1-agents are less likely to be aware of any cholera cases in their neighborhood. Therefore, they will use the usual water fetching point, causing more individuals to be infected with cholera. As the speed of information exchange increases, agents learn from the experience of a larger group of individuals with respect to safety of alternative water fetching points and potential preventive behaviors. Since communication with neighbors is not the sole information source influencing the formation of RP in intelligent BN1-agents, the relation between the number of daily contacts and the resulting number of infected is non-linear: when interaction intensity changes from 7 to 15 people, the number of disease cases decreases by only 25% (Table 4).

#### Sensitivity analysis with respect to the timing of media broadcasting

During the 2005 cholera epidemic in Kumasi, the media began to widely broadcast epidemic information 21 days after the first infected case. We test the sensitivity of risk perception dynamics and the number of infected in response to the different media broadcasting timings. Thus, we ran the Cholera ABM with different media activation dates—10, 30, and 40 days post-infection—in addition to day 22 (the base case of Exp2). Figure 7b illustrates that, generally, when the media reports on the cholera outbreak, the number of BN1-agents perceiving risk increases abruptly. This is true for the media activation scenarios on day 22, 30, and 40; however, this does not hold true for early activation (at day 10). The BN learning algorithm considers several factors at the threat appraisal stage. Thus, although BN1-agents have been alerted about cholera by the media on day 10, they did not yet observe any cholera cases in their household or neighborhood. In addition, depending on the rainfall intensity, they may still have access to safe tap water that will only stop working following heavy rainfall on day 23. This combination of observations within their household and social network triggers BN1-agents to discard media messages and conclude BN1 simulations with low RP.

The timing of media messages does not affect the peak day of an epidemic, but impacts the resulting number of infected individuals (Table 5). It seems that early media attention (day 10) increases public awareness, resulting in individuals taking precautionary measures at a later stage, when other factors contributing to thread appraisal become evident (the yellow RP curve above others at the second half of the epidemics in Fig. 7b). Yet, the relationship is non-linear: the later the announcement, the smaller the marginal impact. Namely, postponing the broadcast for 10 additional days (e.g. day 22 vs. day 30) results in 6% more infected individuals, while another 10 days of delay results in only 2% more infected (day 30 vs. day 40). It is evident that announcing the epidemic 10 days earlier than the base scenario (day 22) reduces infections by over 10%.

**Table 5:** Sensitivity of the extent of an epidemic on the timing of media broadcasting in the population of intelligent agents (Exp2)

Day of media activation	Percentage of total population perceived risk (%)	Epidemic peak day	Percentage of total population infected from the base (%)
Tenth	83.7	36	89.4
Twenty second (base)	100	36	100
Thirtieth	87.8	35	106.1
Fortieth	75.2	35	108.3

### Disease coping strategies: rule-based vs. intelligent risk protection

According to PMT, when individuals are aware of risks, they choose actions based on their response efficacy and self-efficacy (positive influence) and the response costs (negative influence). The population of agents in Exp2 is intelligent in their risk appraisal, but pursue simple, rule-based decision- making (Table 3) at the CA stage.

**Fig. 8:**
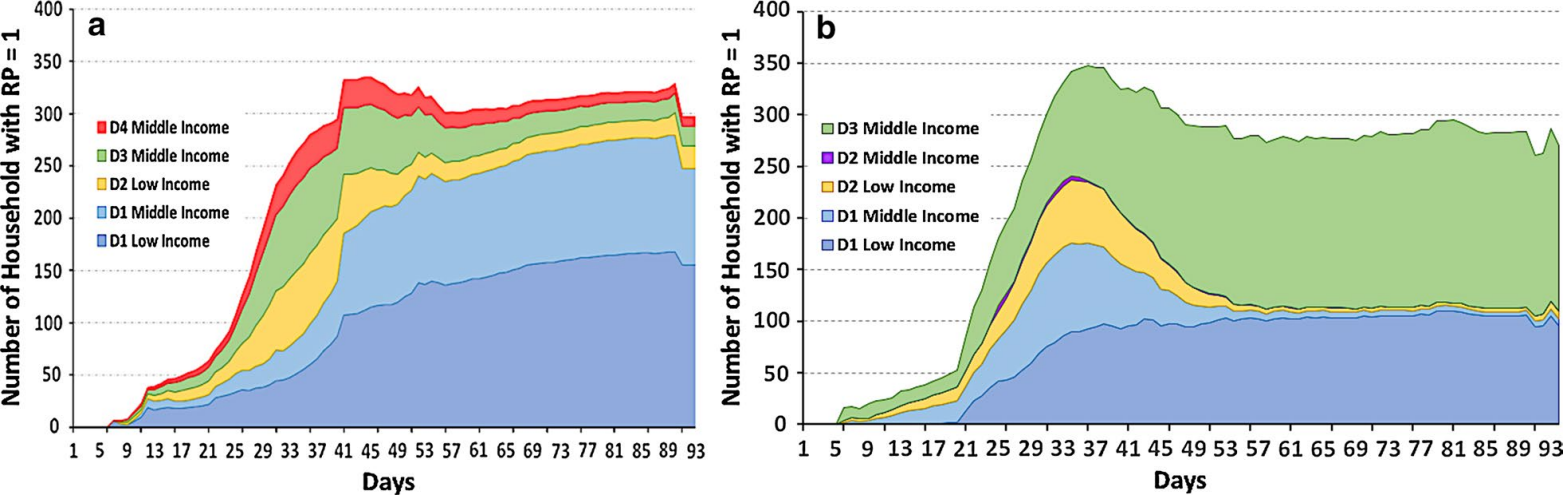
Distribution of preventive actions over time in the population. **a** With deterministic CA decision making (Exp2), **b** with adaptive BN2 CA decision making (Exp3)

Following the heavy rainfall (between days 23 and 50), BN1 agents begin to explore alternative options to drawing water from their normal nearest fetching point (D1). The latter is almost equally chosen by low and middle-income households throughout the entire simulation (Fig. 8a). As cholera risk awareness spreads, the proportion of agents deciding to walk to an alternative fetching point (D2, only low-income households) and to boil water (D3, only middle-income households) increases. Some middle-income households also decide to purchase water (D4). However, since all three alternatives—walk, boil, and purchase—infer additional costs, households shift back to the default D1 option as soon as heavy rainfall ceases, and the number of disease cases decreases. As Fig. 8a. illustrates, a difference also exists in the distribution of preventive actions across income classes. However, the action choice remains deterministic: it depends only on the characteristics of agents at initialization such as income and education. There is no feedback between the effectiveness of previous actions taken by BN1 households or their peers and current agents’ choices regarding water use. Thus, BN1 agents in Exp2 do not learn at the CA stage.

Experiment 3 is run in order explore how the learning process on precautionary measures is reinforced based on previous experiences. Here, agents employ two BN learning algorithms: BN1 for the threat appraisal and BN2 for the CA. When facing cholera risk, agents in Exp3 learn to perceive risk and subsequently learn to protect themselves by making adaptive decisions based on their own previous experience and their neighbors’ experience. The epidemic curves of Exp2 and Exp3 fall within a similar range (Fig. 6b), with one important difference; namely, BN2-agents seem to be over-confident about their disease prevention choices at the epidemic’s onset (approx. day 23), but quickly learn to alter strategies immediately after the peak (Fig. 8b).

Cholera begins to spread from the first few days of the simulation in both Exp2 and Exp3. The total number of infected agents during the cholera epidemic is approximately the same: on average, 14.7% of the simulation’s population (5000 individuals) in both Exp2 and Exp3. However, a qualitative difference exists in the type and dynamics of preventive actions. Figure 8b demonstrates that, over time, agents driven by growing RP learn to boil water based on the previous experience, which leads to a steady increase of D3 strategy use in the BN2 agent population. Among middle and low-income household agents enhanced with BN2, no agents purchase water. Instead, they switch to boiling water (see green D3 zone in Fig. 8b). Simultaneously, the number of middle-income households taking water from their usual, now suspicious-looking fetching point is nearly reduced to zero over time (see the light blue zone in Fig. 8b). BN2-agents also learn that walking to another water collection point still may result in a negative outcome.

**Fig. 9:**
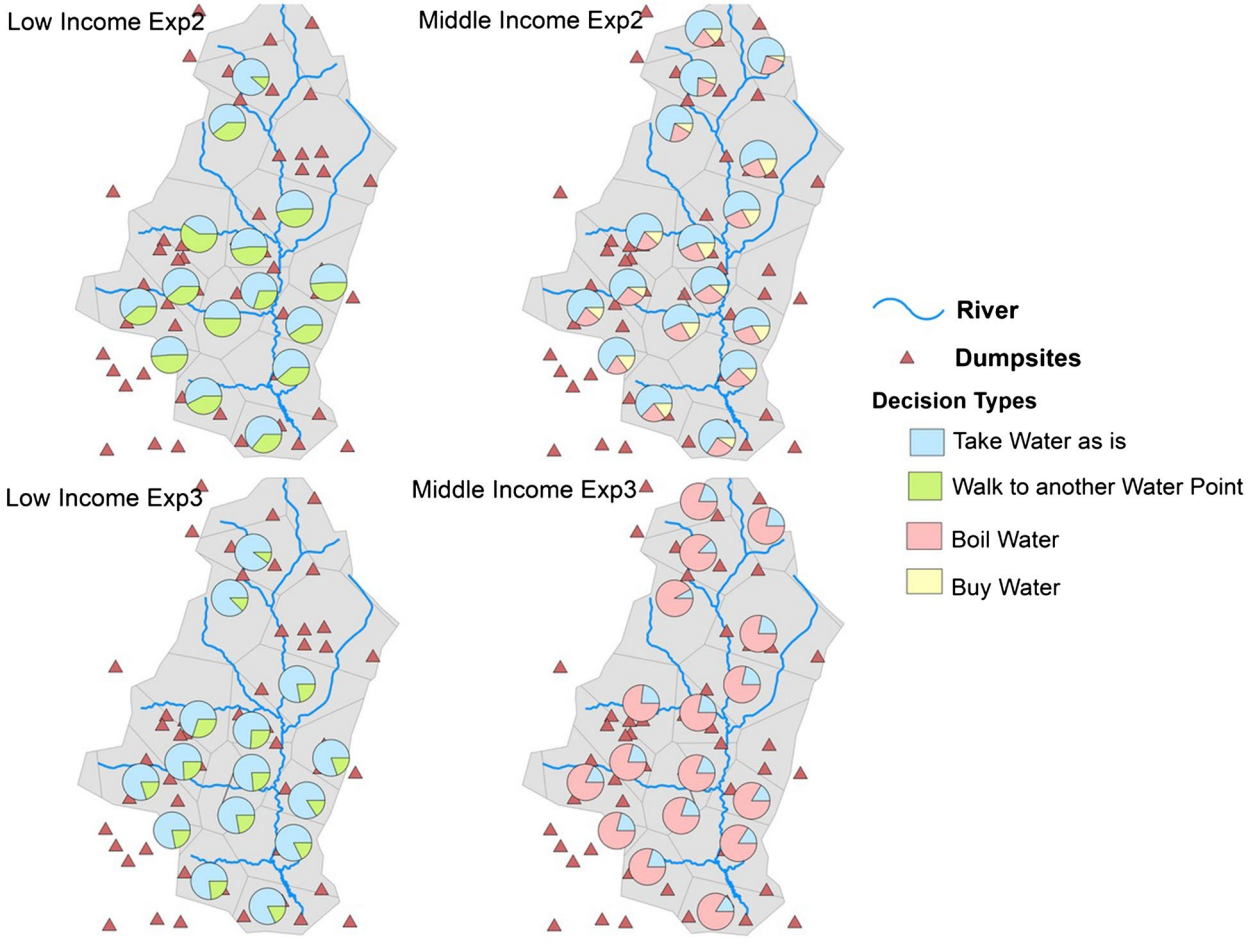
Distribution of preventive actions across space and income groups in Exp2 and Exp3

The distribution of coping strategies between Exp2 and Exp3 also varies in space and by income class (Fig. 9). When low-income BN2-agents learn to compare efficacy and costs based on past experience in Exp3, they realize that walking to another fetching point may not be worth the effort. Instead, in Exp2, low-income agents basing their CA decision on the deterministic rule-based process continue to walk alternate fetching points (compare left-hand side maps in Fig. 9). Non-adaptive middle-income households in Exp2 continue to use a combination of the three strategies provided at initialization. Yet, intelligent BN2 individuals in Exp3 converge to using boiled water in the majority of the cases (right-hand side maps of Fig. 9), as it proved to be most rewarding alternative to D1.

## Conclusions

Risk awareness and risk prevention behavior can have a major impact on the number of disease cases during an epidemic. Models ignoring these elements of human behavior may overestimate the expected number of disease cases. While a number of comprehensive disease ABMs have been developed, few explore the implications of these behavioral aspects and learning. This article introduces an innovative contribution by integrating psychological aspects of decision-making under risk into a spatial ABM using BNs learning algorithms.

We use an empirical spatial ABM of cholera diffusion [19] as a baseline model to test the impact of a multi-stage intelligent decision-making in a risk context. Two sets of BN learning algorithms are designed and coded using R, and are further integrated with the NetLogo-based Cholera ABM. Protection motivation theory from psychology lays the foundation for designing BN learning in two stages: one for RP appraisal and another for coping appraisal. We compare the results of the spatial agent-based disease model without intelligence (zero-intelligence), with an implementation of one-stage BN1 (only RP), and a two-stage BN2 (risk and coping behavior) intelligence. Learning allows a population of heterogeneous and spatially distributed agents to perceive risk and acquire and share knowledge via a social network about the effectiveness of various disease protection actions. This spatial ABM enhanced with BNs allows us to explore the emergence of disease diffusion patterns tracing both geographic, educational, and income inequalities. The implementation strategy, in which we apply both BN1 for risk awareness and BN2 for risk appraisal, seems to outperform an implementation with a single BN. As agents learn about the effectiveness of preventive measures in addition to learning to recognize risks, the society as a whole makes healthier and more cost-effective choices. The sensitivity analysis on the behavioral assumptions indicates that the model is rather robust, with minimal impact on the final outcomes.

While this research presents a step forward in ABMs of disease diffusion by integrating psychology-based intelligence the context of risk, it can be further developed in a number of directions. Firstly, in addition to spatial, hydrological, and socio-economic data, this modeling effort could benefit from disaggregated behavioral data. Currently, our BN1–RP model is updated based on information obtained via personal communication, media, and visual observations of the environment. While we use data from the survey among students from developing countries to parameterize initial weights for RP factors, this may not be fully representative of the population in Kumasi. Disaggregated data on socio-demographic and behavioral characteristics of a target population is in demand to gain better insights on the interplay of factors influencing human behavior during a disease outbreak. This is especially true for visual perception of the environment, as a current lack of information exists on how this factor influences total RP. In addition, a survey to collect data on how media affects people would improve the simulation. Model runs with richer datasets is within the scope of our future work.

Secondly, individual RP and coping appraisal can be implemented in disease ABMs using different ML algorithms. Besides BNs, genetic algorithms or neural networks might also prove useful. Further research is needed to explore the impact of various ML algorithms within the same base ABM. In addition, a systematic study on the performance of one ML algorithm across multiple ABMs for different types of risks in various geographic environments will provide a comprehensive understanding of the implications of introducing intelligence to agent-based modeling will have.

The implementation of risk and coping appraisals in disease ABMs will ultimately aid in supporting decisions regarding the timing of media attention to societal risks, and on the information that must be communicated to the public in order to prevent as many disease cases as possible.

## Appendix 1: Sensitivity analysis of BN1 and BN2

Each parameter (node) in the BNs is associated with a probability (weight). The initial weights for BN1 in Exp2 are estimated from a small sample of data (N = 194) collected within the GeoHealth MOOC in Sep, 2016. To test the impact of the initial weights on the RP in BN1 we performed a simple^1^ sensitivity analysis (Table 6) changing the initial weights of one parameter at a time for VP, media and CNH.

**Table 6:** Sensitivity of the number of agents perceiving risk of cholera (BN1) on the initial weights (probabilities) of the decision nodes (factors)

Nodes with changed weights	Weight	N of agents with RP = 1 (outcome of BN1)	Difference from the base (%)
VP_1_^a^	Low = 1.0, high = 0.0	34,019	110
Base BN1 (Exp2) VP_0_	Default VP: low = 0.8, high = 0.2	30,847	100
VP_2_^a^	Low = 0.6, high = 0.4	30,482	99
Base BN1 (Exp2) M_0_	Default M no = 0.99, yes = 0.01	30,847	100
M_1_^a^	No = 0.79, yes = 0.21	33,174	108
M_2_^a^	No = 0.39, yes = 0.69	33,254	108
CNH_1_^a^	No = 1, yes = 0	31,341	102
Base BN1 (Exp2) CNH_0_	Default CNH no = 0.8, yes = 0.2	30,847	100
CNH_2_^a^	No = 0.6, yes = 0.4	29,519	96

^a^Based on the mean values across 25 runs

Results show that the model behavior is rather robust. Increasing the weight of VP from 0.8 to 1 causes an increase of 10% in the risk perception. Any visual pollution observed will lead to risk awareness. A decrease in the VP weight has no effect.

Changes in the initial weights of media show a change of 8% (Table 6) occurs when the influence of media (both M1 and M2) drop by 20 and 40% correspondingly. While dropping CNH by 40% leads to decrease the number of agents perceiving risk 4%.

BN2 in Exp3 is triggered only when risk is perceived. We checked the sensitivity of the coping strategies of agents with respect to changing the initial weights for the income level in BN2. The shares of agents’ disease coping decisions vary as the probabilities of belonging to a specific income group change (Table 7).

**Table 7:** Sensitivity of the coping strategy choices made by agents with RP = 1 to the initial weights in BN2

Node	Weight	Percentage of decision types of agents with RP = 1			
		D1%	D2%	D3%	D4%
Income_2_^a^	Low = 0.17, middle = 0.83	38	6	49	7
Income	Default: low = 0.27, middle = 0.73	42	7	51	0
Income_1_^a^	Low = 0.37, middle = 0.63	57	24	19	0

^a^The results are based on the mean values from 25 Cholera ABM runs

As income levels in the agent population drops (Income^*^1, 37% increase in the probability of agents with low income level, Table 7), we observe an increase in the uptake of strategies D1 and D2 (drinking water from the river either from the same or an alternative fetching point). If we increase the number of middle-income households, we notice that 7% of the agents start buying water (D4). This explains the decrease in D3 (compared to the original 51%).
